# Surveillance of Human Astrovirus Infection in Brazil: The First Report of MLB1 Astrovirus

**DOI:** 10.1371/journal.pone.0135687

**Published:** 2015-08-14

**Authors:** Maria da Penha Trindade Pinheiro Xavier, Filipe Aníbal Carvalho Costa, Mônica Simões Rocha, Juliana da Silva Ribeiro de Andrade, Fernanda Kreischer Bandeira Diniz, Thais Ramos de Andrade, Marize Pereira Miagostovich, José Paulo Gagliardi Leite, Eduardo de Mello Volotão

**Affiliations:** Laboratory of Comparative and Environmental Virology—Oswaldo Cruz Institute, Oswaldo Cruz Foundation, Ministry of Health, Rio de Janeiro, RJ, Brazil; University of California, San Francisco, UNITED STATES

## Abstract

Human astrovirus (HAstV) represents the third most common virus associated with acute diarrhea (AD). This study aimed to estimate the prevalence of HAstV infection in Brazilian children under 5 years of age with AD, investigate the presence of recently described HAstV strains, through extensive laboratory-based surveillance of enteric viral agents in three Brazilian coastal regions between 2005 and 2011. Using reverse transcription-polymerase chain reaction (RT-PCR), the overall HAstV detection rate reached 7.1% (207/2.913) with percentage varying according to the geographic region: 3.9% (36/921) in the northeast, 7.9% in the south (71/903) and 9.2% in the southeast (100/1.089) (*p* < 0.001). HAstV were detected in cases of all age groups. Detection rates were slightly higher during the spring. Nucleotide sequence analysis of a 320-bp ORF2 fragment revealed that HAstV-1 was the predominant genotype throughout the seven years of the study. The novel AstV-MLB1 was detected in two children with AD from a subset of 200 samples tested, demonstrating the circulation of this virus both the in northeastern and southeastern regions of Brazil. These results provide additional epidemiological and molecular data on HAstV circulation in three Brazilian coastal regions, highlighting its potential to cause infantile AD.

## Introduction

Acute diarrhea (AD) remains a major cause of hospitalization and death in children worldwide, associated with almost 9.9% of the 6.9 million deaths among children under 5 years old in 2011 [1]. Among the AD etiologic agents, viruses play an important role and after rotavirus group A (RVA) and norovirus (NoV), human astrovirus (HAstV) represents the third most common virus found in children with AD, and is thought to be involved in 0.5 to 15% of AD outbreaks [2, 3]. HAstV infections are more frequent in children, the elderly and among immunocompromised patients causing blunting of the tips of the microvilli as well as disruption of the intestinal epithelium [2, 4, 5].

Human astrovirus belongs to the *Astroviridae* family and contains single-stranded, positive-sense, polyadenylated RNA 6.2–7.8 kilobases (kb) in length without an envelope, encased within an icosahedral capsid. The genome contains three open reading frames (ORFs) designated ORF1a, ORF1b and ORF2. The two first ORFs encode non-structural proteins, including viral proteinase and RNA polymerase and ORF2 encodes the capsid protein precursor [2, 3].

The *Astroviridae* family contains two distinguished genera: *Mamastrovirus* and *Avastrovirus*. These viruses were originally classified into genera and species based only on the host of origin. Recently, the Astroviruses Study Group, 9^th^ Report ICTV (International Committee on Taxonomy of Viruses), 2011 [6], proposed a classification based on the amino acid sequence, which encodes the capsid polyprotein and represents the most variable region of the genome [2, 3]. In this classification, *Mamastrovirus* genera associated to human disease is subdivided into four divergent species: *MAstV 1* (the classical human astrovirus 1–8), *MAstV 6* (AstV MLB1-3), *MAstV 8* (AstV VA1 and VA3, also known as HMO-C and HMO-B, respectively) and *MAstV 9* (AstV VA2, also Known as HMO-A, and VA4) [2].

In general, HAstV-1 is the most common type found in children, but the predominant genotype can vary with time and location [3, 7, 8]. The recent identification of novel AstV in humans using highly sensitive methods [9–13], highlights the need to analyze the prevalence of these viruses to recognize their actual impact in public health, since they were found to be genetically related to animal viruses and some were isolated from patients with more severe diseases, such as encephalitis [14–16].

This study aimed to associate the HAstV infection to Brazilian children under 5 years old affected with AD in three Brazilian coastal regions in a seven years period (2005–2011), providing epidemiological and molecular characteristics of HAstV genotypes. Recently described strains of HAstV were also studied.

## Materials and Methods

### Ethical statement

This study, including consent procedures, was approved by the Ethics Committee of Oswaldo Cruz Foundation (CEP 311/06) and is part of an ongoing official Brazilian Ministry of Health surveillance of enteric pathogens to investigate the viral etiology of AD. In this context, stool samples from AD cases were obtained by request of in and out patients attending health centers, including hospitals and public central laboratories, following outbreaks or sporadic cases of AD. For this study, consent was obtained verbally from the parents or relatives guardians on behalf of the children who were enrolled in this study. Data are maintained anonymously.

### Clinical samples and studied areas

For this study, stool samples of 2.913 patients under 5 years old with negative diagnosis for RVA and NoV were obtained between January 2005 and December 2011 from three different Brazilian coastal regions (northeast, southeast and south) with distinct demographic and environmental scenarios and were selected for HAstV investigation. The population of the southern and southeastern regions is of a higher income compared to that of the northeastern region, where much of the population do not have access to sanitation, and where AD-related mortality is higher [17]. Acute diarrhea was defined when children presented three or more liquid or semi-liquid evacuations in a 24-h period.

### Screening of novel HAstVs from stool samples

To search for novel and recently described HAstVs, 200 stool samples obtained between January and December 2011 from patients under two years old were randomly selected from three different Brazilian coastal regions (northeast, southeast and south). All samples were tested previously and were negative for RVA, NoV and HAstV1–8.

### Statistical analysis

Rates of HAstV positivity and association between the frequency of symptoms and the different age groups were compared using the chi-squared test. Statistical significance was established at *p* < 0.05.

### Nucleic acid extraction and detection

Fecal suspension was prepared in a 10% (w/v) Tris/HCL/Ca^2^+ (0,01M, pH 7.2). RNA Extraction was performed using QIAamp Viral RNA extraction kit (Qiagen) methodology according to the manufacturer’s instructions. Detection of classic HAstV and complementary DNA (cDNA) was synthesized using the Applied Biosystems High Capacity cDNA Reverse Transcription kit (Foster City, Ca, USA) with a previous denaturation step with dimethylsulfoxide for 7 min at 97°C. For classic HAstV detection, a PCR protocol was performed using a set of specific primers that targeted the ORF2 region capsid (Mon 269/270 [0.4 μM of each primer]) in a final reaction mixture of 50 μl in volume, according to the PCR conditions described previously [18].

For novel AstV detection, screening was carried out by amplifying a 409 bp segment of the ORF1b (RNA polymerase) region of the AstV genome with consensus primers SF0073/SF0076 designed with a bias for detection of a highly divergent AstV (Classic HAstV 1–8, MLB1-3 and VA1-3) [13]. Positive samples were sequenced and submitted to BLAST (software) [19] searching and those that indicated MLB1 homology were then subjected to amplification and sequencing of the 402 bp segment of the MLB1 ORF2 specific genome region using primers SF0053 and SF0061 for characterization [13]. All amplifications were performed using SuperScript III One-Step RT-PCR System (Invitrogen), according to the manufacturer´s instructions.

For all PCR reactions, final primers concentrations were of 0.2 μM for both forward and reverse primers, in a final reaction volume of 25 uL. The products were analyzed in a 2% agarose gels and visualized by ethidium bromide staining.

### Molecular sequencing

For the molecular characterization of HAstV strains, PCR products were sequenced in both directions using the ABI Prism 3100 Genetic Analyzer and Big Dye Terminator Cycle Sequencing Kit v. 3.1 (Applied Biosystems, Foster City, CA) with the same primers used for the amplification reactions [13,18]. Centri-Sep columns (Princeton Separations, Foster City, CA) were used to purify the sequencing reaction products, according to the manufacturer’s instructions.

### Phylogenetic analysis

The chromatograms obtained from sequencing reactions were carried out using BioEdit software version 7.1.3.0 [20] for sequence editing. Sequence similarity searches were performed with sequences deposited in GenBank using the basic local alignment search tool (BLAST) software [19]. For phylogenetic analysis, reference sequences available for the same genomic region (ORF2) and size were selected taking into account the standards of each genetic type of human and/or environmental AstV and other similar sequences representing different geographical regions and time periods were also used. Sequences analyzes were performed using MEGA software version 5.1 [21] and multiple sequence alignments were carried out using the ClustalW program. Phylogenetic trees based on nucleotide (nt) sequences were obtained using the neighbor-joining method with Kimura two-parameter model with the bootstrap probabilities of each node calculated using 2,000 replicates. Values above 70 were considered significant and are represented in the trees. The nucleotide sequence data reported in this study is available in GenBank under the accession numbers: KM269039-KM269070, KM408170-KM408171 and KC294576-KC294577.

## Results

### Diagnostic and clinical aspects

The overall HAstV positivity rate among RVA and NoV negative AD cases was of 7.1% (207/2.913). Considering the distinct Brazilian coastal regions, HAstV was detected more frequently in the southeastern (9.2% [100/1.089]) and southern (7.9% [71/903]) regions, compared to the northeastern region (3.9% [36/921]); *p* < 0.001 (Table 1).

**Table 1:** Prevalence of HAstV in three Brazilian coastal regions from 2005 to 2011.

				Years				
Regions	2005	2006	2007	2008	2009	2010	2011	Total
**Northeast (%)**		2/21 (9.5)	17/124 (13.7)	3/101 (3.0)	5/95 (2.6)	6/235 (2.6)	3/245 (1.2)	36/921 (3.9)
**Southeast (%)**	19/241 (7.9)	14/133 (10.5)	37/207 (17.9)	8/91 (8.8)	7/147 (4.8)	15/183 (8.2)	0/87 (0)	100/1.089 (9.2)
**South (%)**	10/72 (13.9)	16/117 (1.7)	13/112 (11.6)	11/163 (6.7)	3/122 (2.5)	16/190 (8.4)	2/127 (1.6)	71/903 (7.9)

Human astrovirus was detected in all age groups analyzed: 82/1.224 (6.7%) in infants aged 1–11 months, 64/1.031 (6.2%) in children aged 12–24 months, 48/549 (8.7%) in subjects aged 2–48 months and 13/109 (11.9%) among patients aged 49–60 months. The prevalence rate tended to be higher among children aged 49–60 months (*p* = 0.054; chi-squared test for linear trend), although there was no statistical significance.

Differences in the frequency of some symptoms were observed between HAstV-positive and HAstV-negative patients (Table 2). The presence of mucus in feces was significantly more frequent in HAstV-positive children, particularly in the 1–11-month age group (*p* = 0.012).

**Table 2:** Clinical manifestations of HAstV cases according to age group.

Age Group					
Clinical Characteristics	0–11 months Samples/Studied (%)	12–24 months Samples/Studied (%)	25–48 months Samples/Studied (%)	49–60 months Samples/Studied (%)	Total Samples/Studied (%)
**Fever**					
HAstV negative	716/1233 (58.1)	551/1000 (55.1)	272/505 (53.9)	50/90 (55.6)	1238/2828 (43.7)
HAstV positive	53/85 (62.4)	34/62 (54.8)	21/46 (45.7)	5/9 (55.6)	113/202 (55.9)
***p*-value**	0.438	0.968	0.285	1.000	0.962
**Vomit**					
HAstV negative	634/1235 (51.3)	551/1009 (54.6)	306/509 (60.1)	46/88 (52.3)	1537/2841 (54.1)
HAstV positive	41/84 (51.2)	31/62 (50.0)	26/47 (55.3)	8/10 (80.0)	108/203 (53.2)
***p*-value**	0.986	0.479	0.521	0.094	0.976
**Dehydration**					
HAstV negative	190/557 (34.1)	141/498 (28.3)	63/245 (25.7)	12/53 (22.6)	406/1353 (30)
HAstV positive	15/49 (30.6)	10/35 (28.6)	4/29 (13.8)	2/6 (33.3)	31/119 (26.1)
***p*-value**	0.619	0.973	0.157	0.559	0.365
**Mucous in feces**					
HAstV negative	179/586 (30.5)	132/507 (26.0)	57/251 (22.7)	11/53 (20.8)	379/1397 (27.1)
HAstV positive	25/53 (47.2)	15/38 (39.5)	10/31 (32.3)	1/6 (16.7)	51/128 (39.8)
***p*-value**	**0.012**	0.071	0.238	0.813	**0.002**
**Blood in feces**					
HAstV negative	192/1156 (16.6)	132/948 (13.9)	84/480 (17.5)	6/83 (7.2)	414/2667 (15.5)
HAstV positive	10/81 (12.3)	8/58 (13.8)	4/46 (8.7)	0/10 (0.0)	22/195 (11.3)
***p*-value**	0.315	0.977	0.126	0.379	0.111
**Parenteral fluid reposition**					
HAstV negative	223/345 (64.6)	165/276 (59.8)	82/144 (56.9)	18/31 (58.1)	488/796 (61.3)
HAstV positive	15/25 (60.0)	15/20 (75.0)	5/12 (41.7)	3/4 (75.0)	38/61 (62.3)
***p*-value**	0.170	0.292	0.521	0.735	0.933

**Fig 1:**
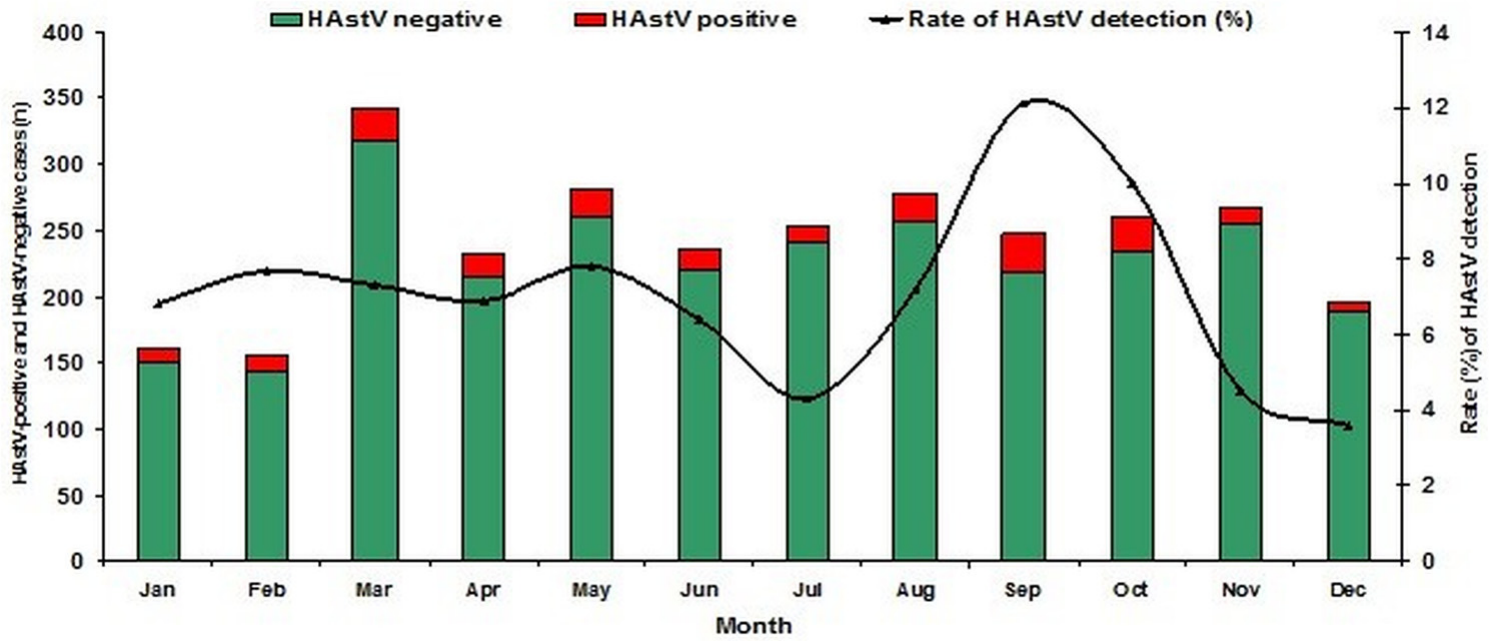
Seasonality of HAstV in cases of acute diarrhea. **Laboratory-based HAstV in three Brazilian regions, between 2005 and 2011.** The rate of HAstV detection by month and number of HAstV-positive samples. Each month represents cumulative observations.

Positivity for HAstV was detected throughout the whole year and seasonally was of 30/513 (5.8%) from January to March, 63/857 (7.4%) from April to June, 46/767 (6.0%) from July to September and 68/776 (8.8%) from October to December (Fig 1).

### Molecular Characterization

Phylogenetic analysis of a 320-bp nucleotide sequence (ORF2) revealed that almost all HAstV genotypes (except HAstV-5 and HAstV-7) circulated in these three Brazilian coastal regions during the studied period. Considering HAstV-positive sequenced strains, HAstV-1 was the most frequent genotype identified, characterized in 77.7% strains.

**Fig 2:**
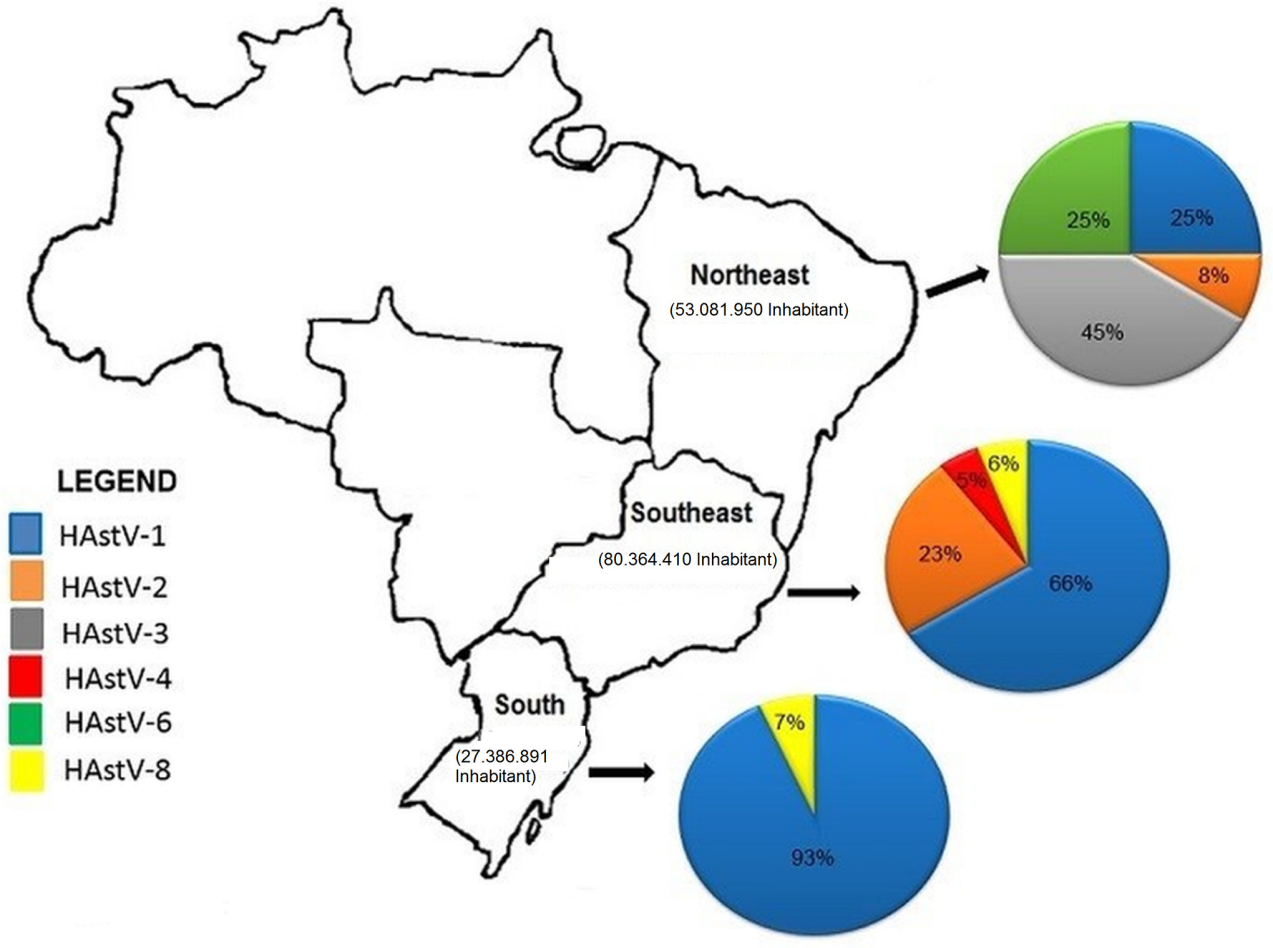
Map of the Brazilian coastal regions, showing the HAstV genotype distributions during 2005–2011.

In all regions studied, HAstV-1 was detected and represented 25% of the strains observed in the northeastern, 66% in the southeastern and 93% in the southern of Brazil. Other genotypes were also observed, including HAstV-3, which represented 45% of the strains detected in the northeastern, followed by HAstV-6 (25%) and HAstV-2 (8%). In the southeastern, the HAstV genotype-2 (23%) and HAstV-8 (6%) and HAstV-4 (5%) were recorded. In the southern region, besides HAstV-1 (93%), HAstV-8 was the only genotype detected (7%) (Fig 2).

**Fig 3:**
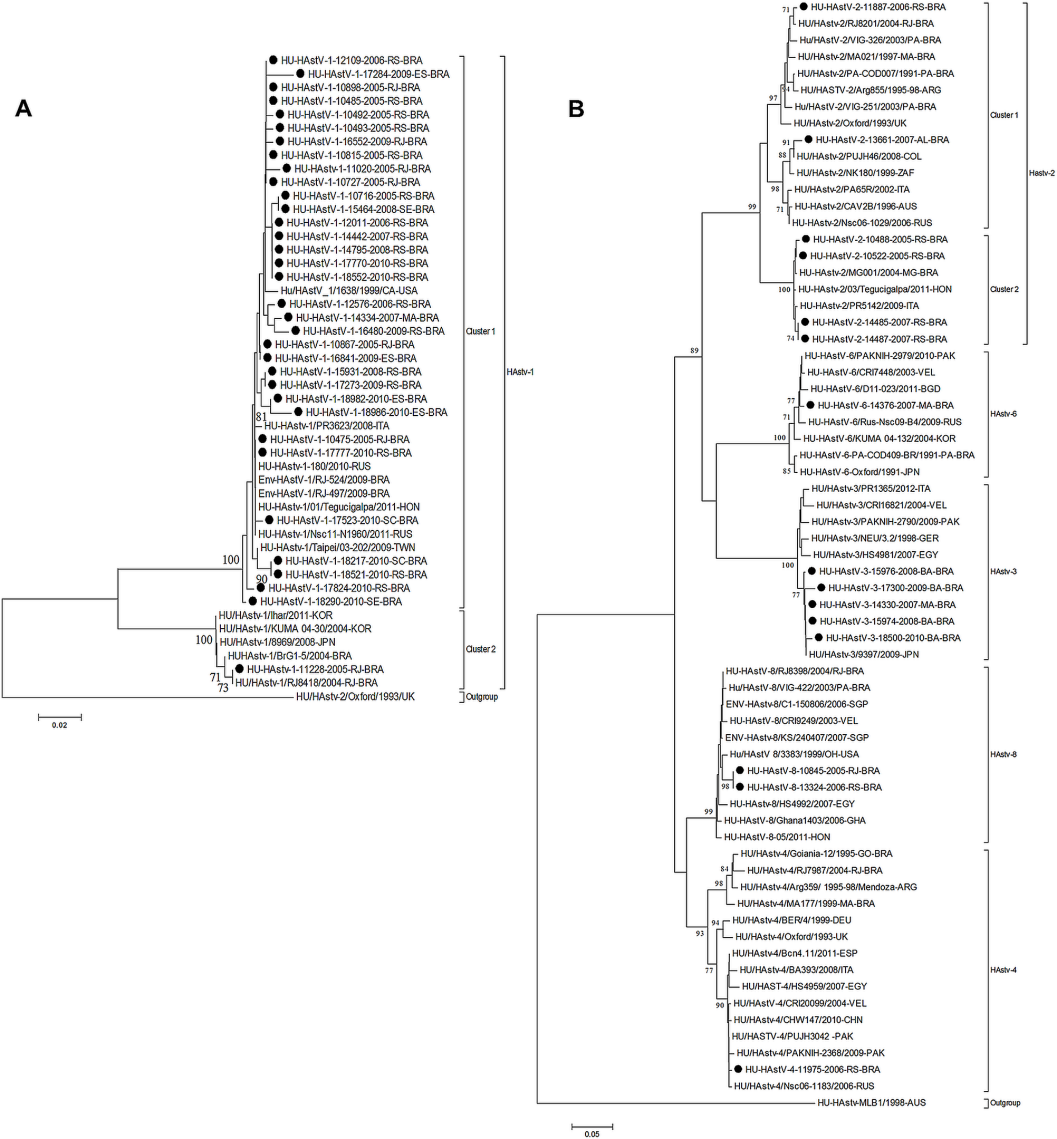
Phylogenetic analyses based on the nucleotide sequences (ORF2 region) of classic HAstV. (A) Phylogenetic analyzes were based on the 320-bp capsid gene between HAstV-1 strains detected in this study and HAstV-1 worldwide strains. (B) Phylogenetic analyzes between strains HAstV-2-HAstV-8 detected in this study and HAstV worldwide strains. The Brazilian strains described in this study are denoted by black circles. The scale bar indicates nucleotide substitutions per site. Bootstrap values (2.000 pseudo-replicates) above 70 are shown. The HAstV reference strains included in this analysis were obtained from GenBank.

Brazilian HAstV-1 strains grouped into two different genetic clusters (Fig 3A). Cluster 1 strains were closely related to each other (98.4–100% identity at the nt level) and were detected in all studied periods in different geographic regions of Brazil and displayed the highest identity (97.8–100%) to worldwide 1999–2011 strains. Cluster 2 grouped Brazilian strains detected from 2004–2005 and some Asiatic 2008–2011 strains were restricted to the southeastern region of Brazil. Brazilian HAstV-2 strains also grouped into two different clusters (Fig 3B). Cluster 1 strains were closely related to each other (96.8–100% identity at the nt level) and were detected in 2006–2007 in different geographic regions (south and northeast), and displayed the highest identity (97.8–100%) to worldwide strains. The second HAstV cluster grouped Brazilian strains detected in 2005 and 2007 together with other Brazilian strains, as well as with Italian and Honduran strains, restricted to the southeastern Brazilian region. The Brazilian HAstV-3 strains showed the highest nucleotide identity (>97%) to HAstV-3 strains reported in Japan, Italy, Pakistan and Egypt. Notably, the unique HAstV-4 strain characterized in this study showed the lowest genetic relationship (93.1–93.7%) with Brazilian samples previously described and the highest nucleotide identity observed with worldwide samples (>96%).

### Screening of novel HAstVs

**Fig 4:**
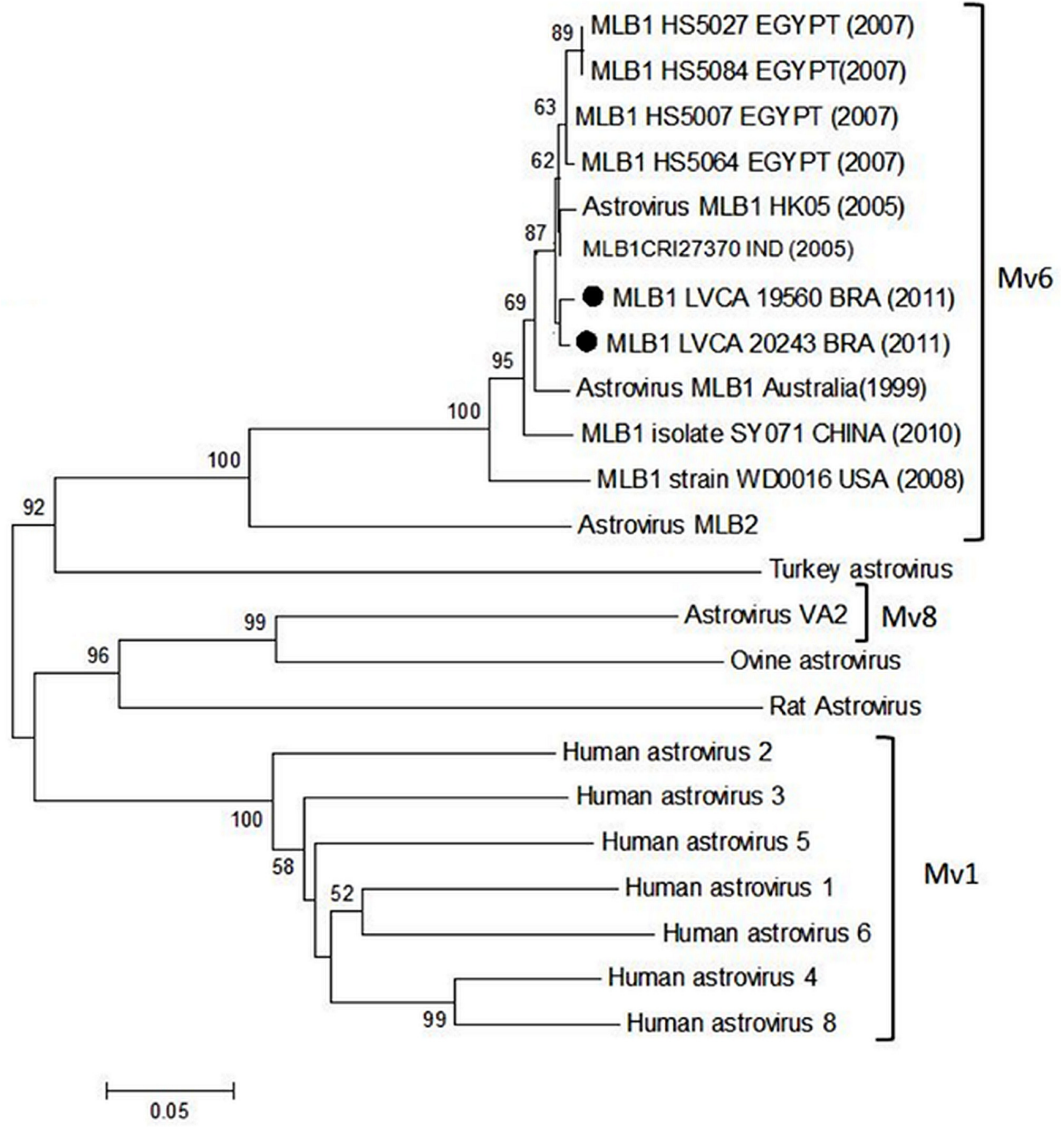
Phylogenetic analyses based on the nucleotide sequences (ORF2 region) of MLB1 Brazilian isolates and representative *Mamastrovirus* species worldwide. The Brazilian strains described in this study are denoted by black circles. The scale bar indicates nucleotide substitutions per site. Bootstrap values (2.000 pseudo-replicates) above 70 are shown. Reference sequences accession number in GenBank Database are cited in order of appearance: NC_011400; FJ402983; NC_014320; JN871245; KC294576; KC294577; HQ674649; HQ674647; HQ674648; HQ6746450; JQ086552; NC_016155; GQ502193; NC_002469; NC_001943; L13745; GU732187; DQ070852; DQ028633; GQ901902; AF260508; HM450382; to Turkey AstV (NC_002470).

Concerning novel HAstV screening, two samples were positive and characterized as AstV-MLB1 (Fig 4). AstV- VA1 or other unusual HAstV rather than MLB1 were not detected. The two positive MLB1 samples were collected from a one year old child admitted in casualty due to AD in February and November 2011, in two different Brazilian regions: Maranhão State (northeast) and Rio de Janeiro State (southeast).

## Discussion

This study reports an overview of the distribution of HAstV genotypes in three coast regions of Brazil. It was the first time that the recently novel HAstV was investigated, increasing knowledge about its characteristics and genotypes circulation. Regarding HAstV infection prevalence in Brazil, initial studies conducted in different regions yielded positivity rates of 3–5% [22–24], lower than those reported in the present survey. Nevertheless, our data showed similar rates to those in other studies carried out in Brazil [8, 25–27] probably due to the evolution of molecular detection methods with increased sensitivity improving diagnosis. High HAstV positivity rates is normally described in AD outbreaks, such as in a native Brazilian population and in southeastern Mexico, where the prevalence of HAstV infection reached 56% and 28%, respectively [28, 29].

As expected, HAstV were commonly detected in all age group analyzed. The prevalence rate observed tended to be higher among children aged 40–60 months, although there was no statistical significance between age group and HAstV infection. HAstVs affect predominantly the pediatric population and the age of children infected with classic HAstV varies, ranging from newborns to over 5 years old [2, 8, 30].

HAstV-related AD is generally considered mild and self-limiting, but it can be sufficiently severe to require medical intervention in immunocompromised or malnourished patients [2, 4, 14, 15, 31]. All children enrolled in this study showed signs and symptoms of AD requiring at least one outpatient visit, thus the HAstV detection in AD cases remains of clinical importance. Fever and vomiting were the most common signs and interestingly, mucus in the stool samples were observed and their presence was statistically significant in HAstV AD cases, mainly in infants aged < 1 year. Data were similar to another study conducted in Brazil [8].

Concerning seasonality, it has been proposed that the peak of HAstV detection in temperate climate countries occurs in the colder months and in tropical areas, the maximum incidence of HAstV infections tends to occur in the rainy season (2). The current study was performed in a seven years’ time span, and a slight trend towards higher detection rates during the rainy season was observed. Similar to our data, the detection of HAstV in tropical climate countries was more frequent in the rainy season [29, 32]. Previous studies carried out in Brazil showed no seasonality or a higher detection frequency in warmer months [8, 24, 25], but the seasonal HAstV pattern is controversial and depends on the climate, geographical region analyzed and the year of study.

Our data demonstrate the variability of circulating HAstV genotypes and that most of the samples were characterized as HAstV-1, reinforcing data from other surveys from several regions of the world, including Brazil that reported the predominance of HAstV-1 [7, 8, 24–26, 33–40]. Phylogenetic analyses showed that Brazilian strains share high nucleotide identity with global strains described in different countries and continents, demonstrating that the introduction of strains occurs continuously and has a great impact on local and regional epidemiology.

The recent discovery from humans of novel AstV strains that show genetic similarities to animal strains has aroused interest and the perception that these potentially zoonotic agents might have a greater impact on public health and on the etiological profile of AD [9–12, 41]. In Brazil, the diagnosis of novel and classic HAstV are restricted to research center, not available in laboratories routine and their importance as etiological agents of AD in both sporadic cases and outbreaks remains poorly understood.

Our investigation of novel HAstV species was conducted in 200 stool samples from children under two years old. The samples tested were selected at random from 2011 cohorts, with equal numbers tested previously negative for RVA, NoV and classic HAstV 1–8 taken from each Brazilian coastal region. This strategy allowed the identification of AstV-MLB1 in two patients. The unusual AstV-MLB1 strain has been identified in fecal samples of patients in Australia and in countries in North America, Asia, Africa and Europe [9, 10, 42–45]. Temporal and geographical relationships were revealed following phylogenetic ORF2 nucleotide analyzes homology among AstV-MLB1 strains (Fig 4). In this study, no other unusual AstV could be detected. Similar results were observed in other surveillance studies [9].

AstV-MLB1 is not yet a well-established AD agent and its role in human health remains unknown. A recent control case study conducted in India could not determine the association between AstV-MLB1 and AD [43]. Another important point is the fact that a large number of samples remain without an etiologic agent, drawing attention to other pathogens that may be involved. Other enteric viruses such as Adenovirus or Sapovirus, as well as bacteria and parasites may also be responsible. This study reinforces the role of distinct HAstV genotypes in the etiological AD profile in different Brazilian coastal regions, describing the detection of AstV-MLB1 in Brazil for the first time and suggesting potential changes in the etiological AD profile considering new pathogens agents as well as novel AstV.
